# Species-level quantification of Faecalibacterium spp. in faeces of healthy Japanese adults

**DOI:** 10.1099/jmm.0.002019

**Published:** 2025-05-23

**Authors:** Masahiro Hirasaki, Ren Kadowaki, Adeline Ang, Gaku Harata, Kenji Miyazawa, Shintaro Maeno, Miguel Gueimonde, Akihito Endo

**Affiliations:** 1 Department of Nutritional Science and Food Safety, Faculty of Applied Bioscience, Tokyo University of Agriculture, 156-8502 Tokyo, Japan; 2 Technical Research Laboratory, Takanashi Milk Products Co., Ltd., 241-0021 Kanagawa, Japan; 3 Research Center for Advanced Science and Innovation, Organization for Research Initiatives, Yamaguchi University, 753-8515 Yamaguchi, Japan; 4 Department of Microbiology and Biochemistry of Dairy Products, IPLA-CSIC, Paseo Rio Linares s/n, 33300 Villaviciosa, Spain

**Keywords:** *Faecalibacterium*, gut microbiota, healthy human adult, quantification, *rpoA*-based qPCR

## Abstract

*A corrigendum of this article has been published full details can be found at* *https://doi.org/10.1099/jmm.0.002043*

*Faecalibacterium prausnitzii* has been considered one of the predominant microbes in the gut microbiota of healthy human adults. Moreover, due to its beneficial metabolites and its reduced population in patients with various disorders, this organism has been regarded as one of the key gut microbes in human health. However, following recent revisions in the taxonomy of the genus *Faecalibacterium* and *F. prausnitzii*, the reported population distribution and health benefits of this species have become unclear. In the present study, the population of nine species-level taxonomic groups (hereafter referred to as species) within *Faecalibacterium* was quantified at the species level in the faeces of healthy Japanese adults (*n*=88). qPCR, combined with *rpoA*-based species-specific primers, showed that *Faecalibacterium taiwanense* had the highest detection rate (prevalence) and copy number among *Faecalibacterium* spp., followed by *Faecalibacterium longum*, *Faecalibacterium duncaniae* and *F. prausnitzii*, while the remaining five species were detected only occasionally. The population of *F. duncaniae* varied significantly between age groups, being higher in individuals in their 40s and 50s compared to those in their 20s (*P*=0.047 and 0.002, respectively). The present study indicates that *F. prausnitzii* is not the predominant *Faecalibacterium* species in the healthy Japanese adults included in the present study. Future studies will shed light on the health benefits of the dominant *Faecalibacterium* spp.

## Introduction

*Faecalibacterium prausnitzii* has been considered one of the predominant microbes in the gut of healthy human adults [1]. It produces butyrate as its primary end product from carbohydrate metabolism, which exhibits multiple beneficial properties, including anti-inflammatory effects, maintenance of gut barrier function and regulation of gut immune homeostasis, contributing to host health through several pathways [2,3]. A reduced population of this microbe has been reported in the gut of patients with various disorders, including Crohn’s disease, ulcerative colitis, type 2 diabetes, mild cognitive impairment and allergic asthma [4,8]. Consequently, this organism has been regarded as a promising biomarker for healthy gut microbiota and a potential next-generation probiotic [1,9]. However, the taxonomical classification of this species, and of the genus *Faecalibacterium* in general, has been recently changed, and the importance of this specific microbe in host health remains unclear.

The genus *Faecalibacterium* was described in 2002 with a single species, *F. prausnitzii* [10], and for nearly two decades, the genus consisted solely of this species. However, several studies suggested genomic heterogeneity of *F. prausnitzii* during this period [11,14]. In 2023, Tanno *et al*. proposed nine species-level taxonomic groups (hereafter referred to as species) within *Faecalibacterium* spp. (including *F. prausnitzii*) found in the human gut, based on genome-based similarity analysis [14,15]. Five of the nine groups were recently (re)classified as *Faecalibacterium butyricigenerans*, *Faecalibacterium duncaniae*, *Faecalibacterium hattorii*, *Faecalibacterium longum* and *Faecalibacterium taiwanense* [16,18]. This (re)classification/division of *F. prausnitzii* has led to debate regarding its predominant population and its reported importance in human health.

To address these concerns, *rpoA*-based uantitative PCR (qPCR) was recently developed to quantify *Faecalibacterium* spp. at the species level [15]. A previous study using this method quantified *Faecalibacterium* spp. in a small number of healthy adults (*n*=6) and reported that *F. prausnitzii* was quantified in only a limited number of subjects. Instead, *F. taiwanense* (described as Group 3 in the study), *F. longum* and *F. butyricigenerans* were more commonly detected in the tested subjects. These findings suggest that these three species are more abundant in healthy adults than *F. prausnitzii* and may be associated with host health. However, the study was preliminary and involved a limited number of subjects.

In the present study, to confirm the population distribution of *Faecalibacterium* spp. at the species level in healthy individuals, faecal samples obtained from 88 healthy Japanese adults were analysed using *rpoA*-based qPCR. The population of *Faecalibacterium* spp. was statistically examined in relation to the basic background information of the subjects.

## Methods

### Subjects and samples

The study was approved by the Ethics Committee of the Shinkohkai Med. Corp., Japan, and conducted in accordance with the Declaration of Helsinki. All subjects were fully informed about the purpose of the study, and a detailed procedure was provided. Written informed consent was obtained from all participants. This study was registered in the UMIN Clinical Trial Registry as UMIN000025888.

In total, 96 healthy Japanese adult volunteers (22 males and 74 females, aged 20–59 years) were initially recruited between January and February 2017. However, eight volunteers were excluded from the present study due to limitations in sample availability. Faecal samples were collected immediately after defecation into plastic containers, transported to the Technical Research Laboratory, Takanashi Milk Products Co., Ltd., at −20 °C, and stored at −80 °C until analysis. These 88 volunteers were not visiting a hospital and were free from subjective symptoms of diseases and severe pollinosis [19]. The basic physiological characteristics of the subjects are presented in Table 1. Stool consistency in the present study ranged from type 2 to type 6 based on the Bristol Stool Form Scale; types 1 and 7 were not included. A previous study reported that stool water content does not vary markedly among different consistencies (e.g. hard and formed, soft but formed and loose and unformed) [20].

**Table 1.** Physiological characteristics of the subjects (*n*=88)

Sex		Male	22 (25 %)
		Female	66 (75 %)
Age		20 s (20<X<29)	17
	median (Q1-Q3)	30 s (30<X<39)	20
	=41.5(32.75–50)	40 s (40<X<49)	27
		50 s (50<X<59)	24
BMI		<20	39
	median (Q1-Q3)	20<X 25	40
	=20.4(18.21–22.57)	25<X<30	7
		>30	2

### DNA extraction

DNA was extracted from faecal samples using the NucleoSpin^®^ DNA Stool kit (MACHEREY-NAGEL GmbH and Co., KG, Düren, Germany) following the manufacturer’s instructions within 1 month of sample receipt. The DNA concentration and quality were assessed using a Qubit 3.0 fluorometer (Invitrogen, Waltham, MA, USA).

### 16S rRNA gene sequencing

The sequencing library was prepared using index-appended amplicons of the V3–V4 region of the 16S rRNA gene according to the 16S Metagenomic Sequencing Library Preparation manual (Illumina, San Diego, CA, USA). Pair-end sequencing was performed on the MiSeq platform (Illumina) using a MiSeq Reagent Kit v3 (600 cycles) (Illumina). Data analysis was conducted using QIIME2 (v.2024.5) with default settings. A total of 9,885,007 sequence reads were obtained (average±sd per samples=56,164±23,803), which were imported into QIIME2. Quality assessment, filtering, barcode trimming and chimaera detection were performed using the DADA2 pipeline (v1.30.0), resulting in a total of 6,777,082 high-quality reads. Taxonomic classification was assigned to amplicon sequence variants using the SILVA database (release 138) with taxonomic classification at >99% confidence. Sequencing data were deposited in the DDBJ Sequence Read Archive under the accession numbers DRR657747–DRR657922.

### Quantification of *Faecalibacterium* spp.

Quantification of *Faecalibacterium* spp. was performed as described previously [15]. FastStart Essential DNA Green Master Mix combined with the LightCycler 96 system (Roche, Basel, Switzerland) was used for qPCR according to the manufacturer’s instructions. All primers used in this qPCR study are listed in Table S1, available in the online Supplementary Material. The nine species within *Faecalibacterium*, whose classification is summarized in Table 2, were quantified using nine primer pairs specific to the *rpoA* gene of each species [15]. The *rpoA* gene is among the housekeeping genes of bacteria, and all *Faecalibacterium* genomes contain a single copy of the *rpoA* gene [15]. The qPCR programme consisted of an initial denaturation at 95 °C for 10 min and 45 cycles of 95 °C for 10 s, 60 °C for 10 s and 72 °C for 15–25 s (Table S1). Standard curves were generated for each primer pair using synthetic *rpoA* gene DNA of the targeted species, described in a previous study [15]. In this study, *F. gallinarum* was not included due to its distinct origin (i.e. chicken) [16]. Additionally, *Faecalibacterium wellingii*, which was only recently validated (validated in January 2025) [21], was not included in the present study.

**Table 2.** Prevalence (%) and population [median and IQR (Q1–Q3) of log_10_ copies g^−1^ of faeces] of *Faecalibacterium* genus and each *Faecalibacterium* spp. in healthy Japanese subjects (*n*=88)

	Prevalence (%)	Median (Q1–Q3)	Correlation coefficient*
Genus	97.7	7.81 (7.30–8.35)	0.507
*F. prausnitzii* (Group 1)	72.7	4.92 (BQL–6.13)	0.522
Group 2	6.8	BQL (BQL–BQL)	0.147
*F. taiwanense* (Group 3)	89.8	5.85 (4.77–6.70)	0.583
*F. longum* (Group 4)	77.3	5.48 (4.02–6.30)	0.375
Group 5	34.1	BQL (BQL–4.37)	0.109
*F. duncaniae* (Group 6)	72.7	5.61 (BQL–6.58)	0.475
*F. hattorii* (Group 7)	9.1	BQL (BQL–BQL)	0.247
Group 8	34.0	BQL (BQL–4.47)	0.126
*F. butyricigenerans* (Group 9)	20.5	BQL (BQL–BQL)	−0.061

*Correlation coefficients between the relative abundance of the genus *Faecalibacterium*, as determined by 16S rRNA gene sequencing, and the population of the genus *Faecalibacterium* and each species, as determined by qPCR, are presented.

BQL, below quantification limit (3.43).

Genus-level quantification of *Faecalibacterium* was performed using the primer pair Fprau223F/Fpau420R, specific to the 16S rRNA gene of all *Faecalibacterium* spp., designed by Bartosh and coworkers [22]. The qPCR programme for genus-level quantification consisted of an initial denaturation at 95 °C for 10 min, followed by 45 cycles of 95 °C for 10 s, 58 °C for 10 s and 72 °C for 20 s. A standard curve was generated by using serially diluted synthetic 16S rRNA gene DNA of *F. prausnitzii* ATCC 27768^T^, as described previously [14].

In all qPCR assays, a melting peak analysis was conducted to confirm specific amplification. When melting temperatures of amplicons differed >1.5 °C from that of the reference DNA, the amplification was considered negative. Samples were run in duplicate on the same plate, and the mean and sd were obtained. The quantification limit of qPCR was determined based on serially diluted synthetic DNA. If amplification was not observed or observed but below the quantification limit (BQL), the result was recorded as BQL.

### Statistical analysis

The Mann–Whitney U test was used to compare *Faecalibacterium* spp. populations between two groups, while the Kruskal–Wallis test with Bonferroni correction was applied when comparing three or more groups. A *P* value of <0.05 was considered statistically significant. Statistical analysis was performed using IBM SPSS Statistics for Windows (version 26). The correlation coefficients between relative abundance of *Faecalibacterium* based on 16S rRNA gene sequencing and population of the genus *Faecalibacterium* and each species as determined by qPCR were determined using Spearman’s rank correlation in R (ver. 4.4.2).

## Results

### Microbial composition of faecal samples determined by 16S rRNA gene sequencing

**Fig. 1.**
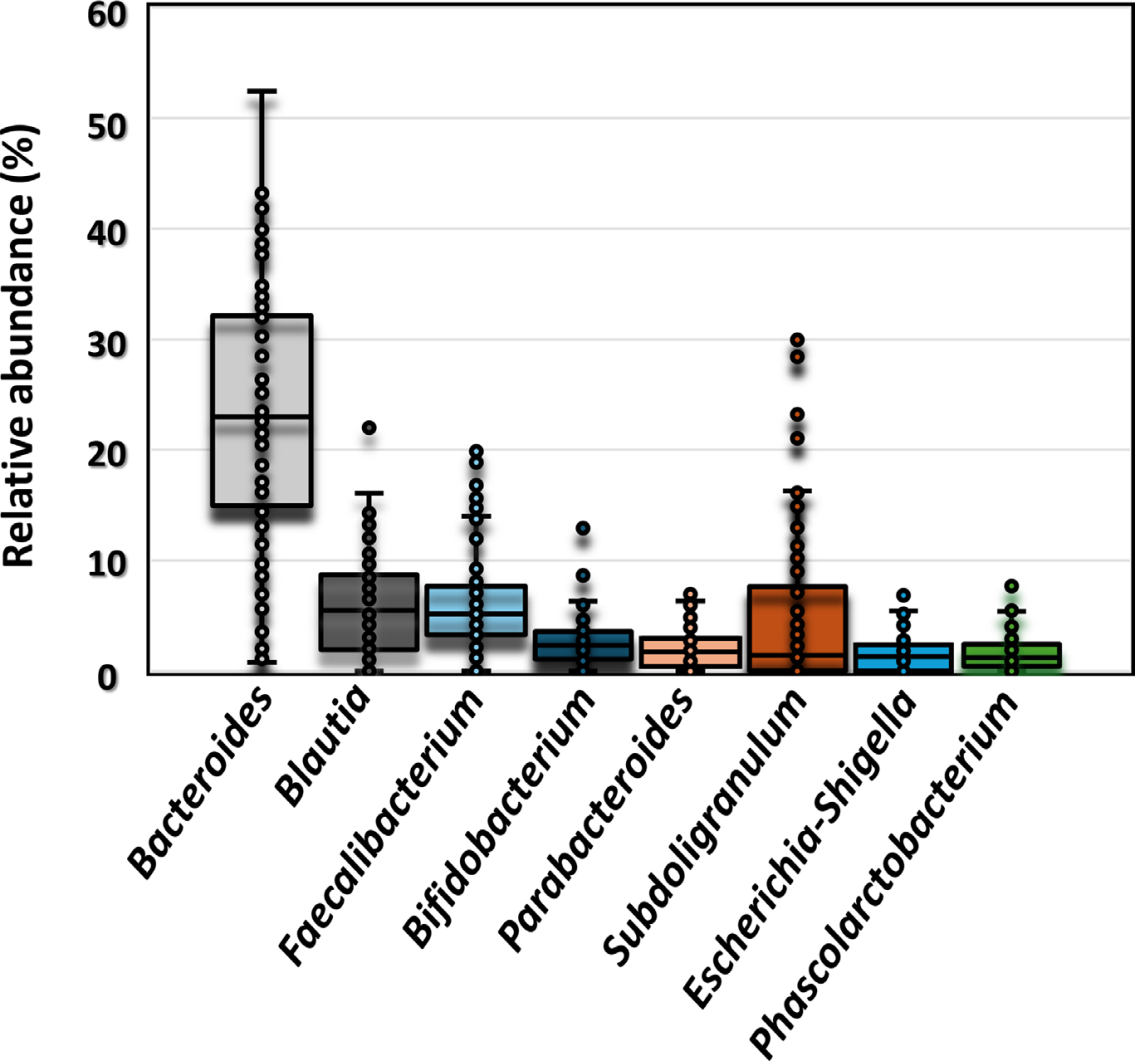
Relative abundance of the top eight genera (median>1%) in healthy Japanese subjects (*n*=88) based on 16S rRNA gene sequencing. The box plots represent the median and IQR, and the dots indicate individuals.

The microbial composition in faecal samples was initially analysed by 16S rRNA gene sequencing to determine the relative abundance of *Faecalibacterium* at the genus level. Among the healthy Japanese subjects tested, the genus *Bacteroides* exhibited the highest relative abundance (median=22.98%), followed by *Blautia* (median=6.28%) (Fig. 1). *Faecalibacterium* ranked third, with a median (interquartile range, IQR, Q1–Q3) of 5.49% (1.97–8.64%).

### Quantification and prevalence of *Faecalibacterium* spp.

Detection and quantification of the *Faecalibacterium* genus were performed using 16S-based qPCR, while species-level quantification was conducted using *rpoA*-based qPCR. Melting peak analysis was performed to confirm the reliability of the amplification. The prevalence of the genus *Faecalibacterium* and individual species is summarized in Table 2. *Faecalibacterium* genus was detected in 97.7% of the subjects. At the species level, *F. taiwanense* (Group 3) was detected in 89.8% of the subjects, and *F. longum* (Group 4), *F. duncaniae* (Group 6) and *F. prausnitzii* (Group 1) were seen in 70–80% of the subjects. Groups 5 and 8, as well as *F. butyricigenerans* (Group 9), were detected in 34.1, 34.0 and 20.5% of the samples, respectively. *F. hattorii* (Group 7) and Group 2 were detected in fewer than 10% of subjects. Non-specific amplification was observed for qPCR of the latter five species with low prevalence, which were considered negative in prevalence analysis.

**Fig. 2.**
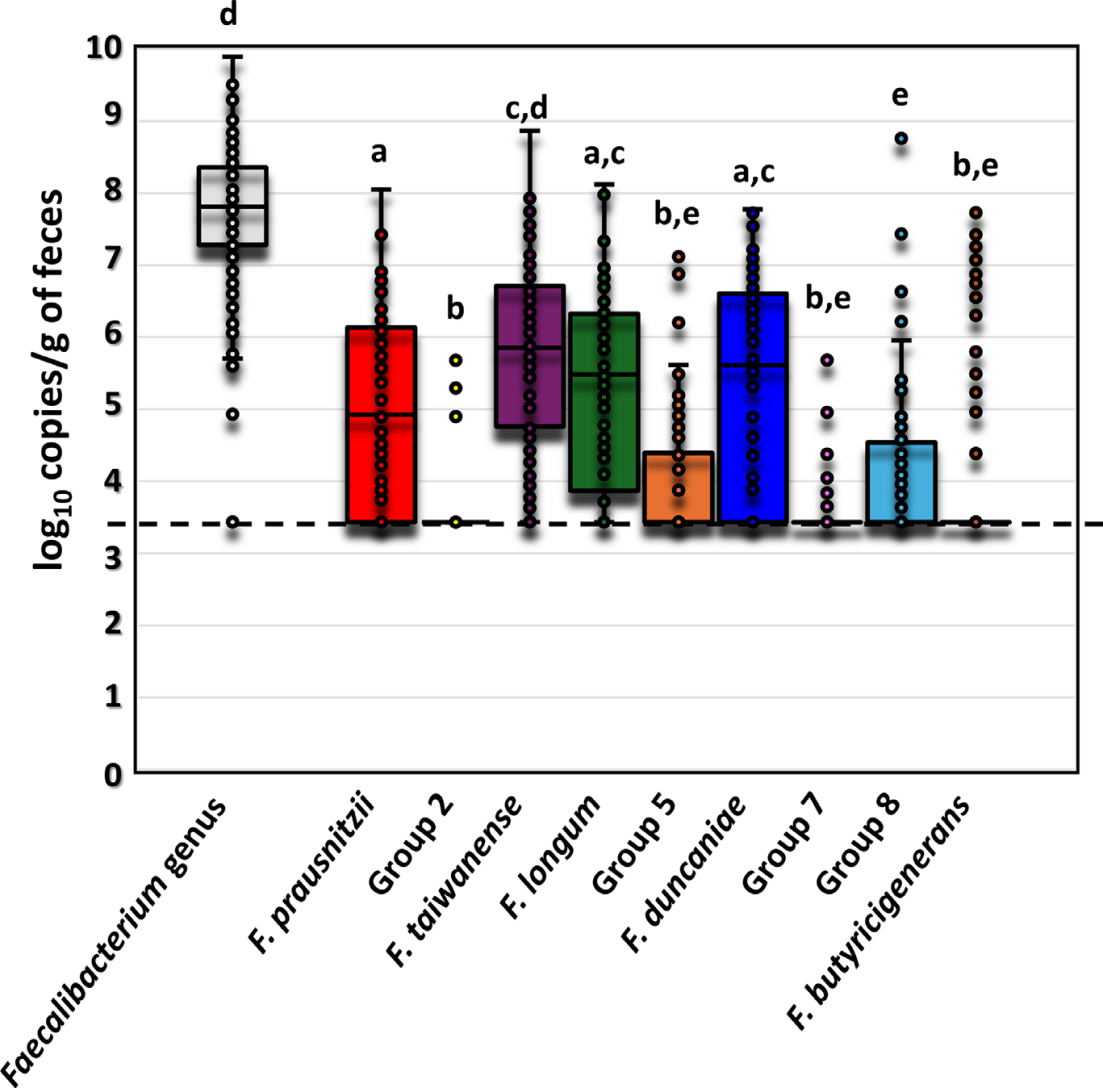
Population (log_10_ copies g^−1^ of faeces) of *Faecalibacterium* genus and each *Faecalibacterium* species in healthy Japanese subjects (*n*=88) as determined by qPCR. The box plots represent the median and IQR, and the dots indicate individuals. Quantification limit (3.43) was indicated with the dashed line. Different letters (a–e) on top of the bars indicate significant differences (*P*<0.05) by the Kruskal–Wallis test with multiple comparisons by the Bonferroni correction.

The median (IQR, Q1–Q3) of *Faecalibacterium* genus-level population (log_10_ copies g^−1^ faeces) in the 88 samples was 7.81 (7.30–8.35) (Fig. 2 and Table 2). Among species, the highest median (Q1–Q3) was recorded for *F. taiwanense* (Group 3) at 5.85 (4.77–6.70), followed by *F. duncaniae* (Group 6) at 5.61 (BQL–6.58), *F. longum* (Group 4) at 5.48 (4.02–6.30) and *F. prausnitzii* (Group 1) at 4.92 (BQL–6.12) (Fig. 2 and Table 2). The medians of *F. hattorii* (Group 7), *F. butyricigenerans* (Group 9) and Groups 2, 5 and 8 were BQL.

Correlation analysis was conducted between the relative abundance of the *Faecalibacterium* genus, as determined by 16S rRNA gene sequencing, and the population of the genus and each species, as determined by qPCR. The results indicate that the population of the genus, *F. taiwanense* (Group 3) and *F. prausnitzii* (Group 1) showed correlation with the relative abundance of the *Faecalibacterium* genus (correlation coefficient >0.5, *P*<0.001) (Table 2). Populations of *F. duncaniae* (Group 6) and *F. longum* (Group 4) showed a weak correlation (correlation coefficient >0.3, *P*<0.001), while the other species showed no significant correlations.

### Population of *Faecalibacterium* spp. across different physiological backgrounds in healthy Japanese subjects

**Fig. 3.**
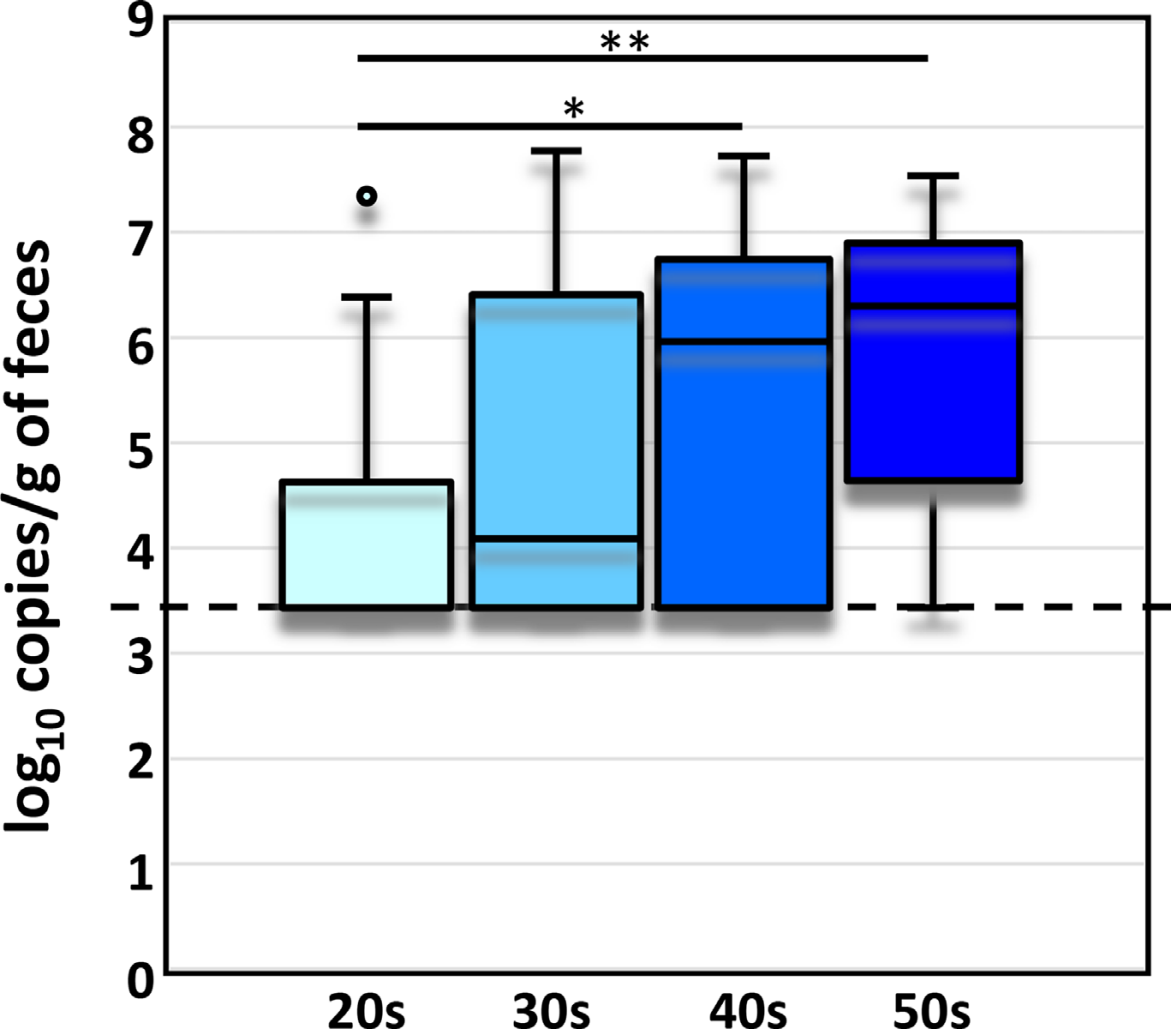
Levels of *F. duncaniae* (Group 6) among different age groups. The box plots represent the median and IQR, and the separated dots indicate outliers. The quantification limit (3.43) was indicated with a dashed line. The Kruskal–Wallis test with multiple comparisons by the Bonferroni correction was used for statistical analysis. ^*^, adjusted *P* value<0.05; ^**^, adjusted *P* value<0.01.

Subjects were grouped based on physiological characteristics, including sex, age and body mass index (BMI), and populations of *Faecalibacterium* spp. were compared among groups. Among different age groups, *F. duncaniae* (Group 6) showed a significant difference in population size (*P*=0.004) (Table 3). The levels of *F. dancaniae* (Group 6) were significantly higher in subjects in their 40s (median, Q1–Q3=5.96, 3.60–6.65; *P*=0.047) and 50s (6.29, 4.69–6.84; *P*=0.002) compared to those in their 20s (BQL, BQL–3.52) (Fig. 3). Similarly, the levels of the *Faecalibacterium* genus, *F. prausnitzii* (Group 1), *F. taiwanense* (Group 3) and *F. longum* (Group 4), exhibited trends towards differences among age groups (*P*<0.1, Table 3), also increasing with age but not reaching statistical significance. No statistically significant differences were found in the population of *Faecalibacterium* spp. between sexes or BMI groups.

**Table 3.** Population [median and IQR (Q1–Q3) of log_10_ copies g^−1^ of faeces] of *Faecalibacterium* genus and each *Faecalibacterium* spp. in different age groups

	Age	Median (Q1–Q3)	Adjusted *P* value
Genus	20s	7.47 (6.61–7.68)	
	30s	7.91 (7.59–8.35)	0.056
	40s	8.89 (7.15–8.56)	
	50s	8.09 (7.50–8.35)	
*F. prausnitzii* (Group 1)	20s	4.27 (BQL–5.12)	
	30s	4.69 (BQL–5.91)	0.097
	40s	4.97 (3.83–6.18)	
	50s	5.48 (3.97–6.36)	
*F. taiwanense* (Group 3)	20s	5.21 (4.51–6.30)	
	30s	5.76 (4.81–6.50)	0.055
	40s	5.59 (4.06–6.55)	
	50s	6.37 (5.79–7.18)	
*F. longum* (Group 4)	20s	5.33 (4.32–5.70)	
	30s	5.62 (4.47–6.34)	0.054
	40s	4.83 (3.50–6.09)	
	50s	6.07 (5.42–6.50)	
*F. duncaniae* (Group 6)	20s	BQL (BQL–3.53)	
	30s	4.08 (BQL–6.37)	0.004
	40s	5.96 (3.55–6.65)	
	50s	6.29 (4.69–6.85)	

Populations of *Faecalibacterium* spp. among different age groups were statistically analysed with the Kruskal–Wallis test with multiple comparisons by the Bonferroni correction.

BQL, below quantification limit (3.43).

## Discussion

Due to recent significant taxonomic changes of the genus *Faecalibacterium* and *F. prausnitzii*, the reported population and specific roles of this species have become unclear. The present study characterized the population of *Faecalibacterium* at the species level in healthy Japanese subjects. The genus *Faecalibacterium* was the third most abundant genus, with a median relative abundance of 5.49%, a level similar to those previously reported in the adult gut [23,24]. qPCR using *rpoA*-based species-specific primers revealed that *F. taiwanense* was the most prevalent and the numerically dominant *Faecalibacterium* species among the subjects, followed by *F. longum*, *F. duncaniae* and *F. prausnitzii*. This is the first study to demonstrate that *F. taiwanense*, *F. longum* and *F. duncaniae*, rather than *F. prausnitzii*, are the predominant *Faecalibacterium* species in the human gut. A previous study using a small number of subjects (*n*=6) suggested this possibility [15], and *F. longum* and * F. duncaniae* showed similar population sizes with *F. taiwanense* but were found at slightly lower prevalence rates. However, it should be noted that the subject groups in both the present and previous studies were not fully representative of the general healthy Japanese adult population due to factors such as gender imbalance. Further studies would help to understand the predominance and significance of *Faecalibacterium* spp. in the population.

A previous study suggested *F. butyricigenerans* as one of the most prevalent *Faecalibacterium* species among six subjects [15], but its prevalence was low in the present study. This discrepancy may be due to non-specific amplification in qPCR of the species found in the present study, suggesting that *F. butyricigenerans* is a minor species in healthy Japanese adults. Indeed, De Filippis *et al*. described 12 *Faecalibacterium* clades (A–L) from the human gut using 2,859 globally collected *Faecalibacterium* genomes, but no clade corresponding to *F. butyricigenerans* was seen [23]. The number of copies per g of faeces found at the species level showed slight discrepancies compared to those at the genus level (Fig. 2 and Table 2). This difference can be attributed to the variation in copy numbers between the 16S rRNA gene, used for genus-level quantification, and the *rpoA* gene, used for species-level quantification, as described previously [15]. In the *Faecalibacterium* genome, the 16S rRNA gene is present in six copies, while the *rpoA* gene is present in a single copy [14,15]. Therefore, the copy number detected at the genus level using the 16S rRNA gene is theoretically six times higher than the sum of the copy numbers detected at the species level using the *rpoA* gene.

Statistical analysis based on physiological background showed significant differences only in *F. duncaniae* across age groups, with higher populations in subjects in their 40s and 50s compared to those in their 20s. The reason for this age-related difference remains unclear, though diet habits may play a role. Further studies are needed to investigate the colonization and development of *Faecalibacterium* species with ageing.

Previous studies have reported *F. prausnitzii* as the dominant gut microbe in healthy human adults and have linked its reduced population in patients with several disorders [7,25]. However, our findings disagreed with its predominant position. *F. taiwanense*, *F. longum* and *F. duncaniae* exhibited higher populations than *F. prausnitzii*. The type strain of *F. duncaniae*, strain A2-165^T^, is the most well-characterized strain among *Faecalibacterium* spp., and its potential health benefits have been reported in previous *in vitro* and *in vivo* studies [26,27]. The importance of *F. taiwanense* and *F. longum* remains poorly understood. Future studies should elucidate the health benefits of these dominant *Faecalibacterium* spp. through *in vitro*, *in vivo* and clinical studies.

## Supplementary material

10.1099/jmm.0.002019 Uncited Table S1.

## Abbreviations

BMI: body mass index
BQL: below the quantification limit
IQR: interquartile range
