# Corrigendum: Species-level quantification of Faecalibacterium spp. in faeces of healthy Japanese adults

**DOI:** 10.1099/jmm.0.002043

**Published:** 2025-07-23

**Authors:** Masahiro Hirasaki, Ren Kadowaki, Adeline Ang, Gaku Harata, Kenji Miyazawa, Shintaro Maeno, Miguel Gueimonde, Akihito Endo

**Affiliations:** 1 Department of Nutritional Science and Food Safety, Faculty of Applied Bioscience, Tokyo University of Agriculture, 156-8502 Tokyo, Japan; 2 Technical Research Laboratory, Takanashi Milk Products Co., Ltd., 241-0021 Kanagawa, Japan; 3 Research Center for Advanced Science and Innovation, Organization for Research Initiatives, Yamaguchi University, 753-8515 Yamaguchi, Japan; 4 Department of Microbiology and Biochemistry of Dairy Products, IPLA-CSIC, Paseo Rio Linares s/n, 33300 Villaviciosa, Spain

In the published version of this article, there was a formatting error in one of the author’s name which resulted in the surname being presented incorrectly.

The correct surname is ‘Ang’, followed by the first name of ‘Adeline’. The official arrangement of full name is Adeline Ang. The paper should therefore be cited as “Hirasaki M, Kadowaki R, Ang A, Harata G, Miyazawa K, Maeno S, Gueimonde M, Endo A. J Med Microbiol. 2025 May;74 : 002019’.

This has been corrected in the author list above and also in the published article.

The authors apologize for any inconvenience caused.

