# Supplementary material for: Species-level quantification of Faecalibacterium spp. in faeces of healthy Japanese adults
Source: J Med Microbiol. 2025 May 23;74(5):002019. doi: 10.1099/jmm.0.002019 (PMC12102495; doi:10.1099/jmm.0.002019)
Supplement: Uncited Table S1. [file jmm-74-02019-s001.pdf]

supplemental Table S1. Primers used in qPCR

Primer	Sequence (5'→3')	Target	References	Time for extension (s)
Fprau223F	GATGGCCTCGCGTCCGATTAG	*Faecalibacterium* genus	Bartosch et al. (2004)	20
Fprau420R	CCGAAGACCTTCTTCCTCC	*Faecalibacterium* genus	Bartosch et al. (2004)	
Faecali-group1-sp-F	CCTGAGTGGCACATTGCAACT	Group 1 (*F. prausnitzii*)	Tanno et al. (2023)	15
Faecali-group1-sp-R	TAAATGCTGTCAACGGGAAGG	Group 1 (*F. prausnitzii*)	Tanno et al. (2023)	
Faecali-group2-sp-F	CCAAGCTCGTCATGGAGCTC	Group 2	Tanno et al. (2023)	20
Faecali-group2-sp-R	ATGGTCAGCTTGTCGTAGTCA	Group 2	Tanno et al. (2023)	
Faecali-group3-sp-F	AACCTGTCCGATGAGGCAGCC	Group 3 (*F. taiwanense*)	Tanno et al. (2023)	15
Faecali-group3-sp-R	TCTTCCACCGTGTTGATGCCT	Group 3 (*F. taiwanense*)	Tanno et al. (2023)	
Faecali-group4-sp-F	GCCATCATCGAGAAGAATGAC	Group 4 (*F. longum*)	Tanno et al. (2023)	15
Faecali-group4-sp-R	TGTGCATTGATCGTGCCATCC	Group 4 (*F. longum*)	Tanno et al. (2023)	
Faecali-group5-sp-F	GAAATTGTCCTGAACCTGAAA	Group 5	Tanno et al. (2023)	25
Faecali-group5-sp-R	CCTGTTTGTTGCGCTCAGCC	Group 5	Tanno et al. (2023)	
Faecali-group6-sp-F	AAGGGCCGCGGTTATGTGCCT	Group 6 (*F. duncaniae*)	Tanno et al. (2023)	15
Faecali-group6-sp-R	TAATCGATGGCCTGTCCAACG	Group 6 (*F. duncaniae*)	Tanno et al. (2023)	
Faecali-group7-sp-F	CCTGAATGGCACATCGCAACCT	Group 7 (*F. hattorii*)	Tanno et al. (2023)	15
Faecali-group7-sp-R	ATGCTATCGACGGGAAGCGTA	Group 7 (*F. hattorii*)	Tanno et al. (2023)	
Faecali-group8-sp-F	GGTGAATTACAATGTTGAGAA	Group 8	Tanno et al. (2023)	20
Faecali-group8-sp-R	TCTCGGTGCCAGCGGCCTCA	Group 8	Tanno et al. (2023)	
Faecali-group9-sp-F	AATGTCGAGAGCACCCGTGTG	Group 9 (*F. butyricigenerans*)	Tanno et al. (2023)	20
Faecali-group9-sp-R	GATCTCAGCGCCAGCGGCCTCG	Group 9 (*F. butyricigenerans*)	Tanno et al. (2023)	

Bartosch S, Fite A, Macfarlane GT, McMurdo ME. Characterization of bacterial communities in feces from healthy elderly volunteers and hospitalized elderly patients by using real-time PCR and effects of antibiotic treatment on the fecal microbiota. Applied and environmental microbiology 2004;70(6):3575-3581.

Tanno H, Chatel JM, Martin R, Mariat D, Sakamoto M, Yamazaki M, Salminen S, Gueimonde M, Endo A. New gene markers for classification and quantification of *Faecalibacterium* spp. in the human gut. FEMS microbiology ecology 2023;99(5):fiad035.
